# Relevance of 3d culture systems to study osteosarcoma environment

**DOI:** 10.1186/s13046-017-0663-5

**Published:** 2018-01-05

**Authors:** Angela De Luca, Lavinia Raimondi, Francesca Salamanna, Valeria Carina, Viviana Costa, Daniele Bellavia, Riccardo Alessandro, Milena Fini, Gianluca Giavaresi

**Affiliations:** 1 IRCCS Rizzoli Orthopedic Institute, Bologna, Italy; 2 IRCCS Rizzoli Orthopedic Institute, Innovative Technologic Platform for Tissue Engineering, Theranostics and Oncology, Via Divisi, 83, 90133 Palermo, Italy; 3 IRCCS Rizzoli Orthopedic Institute, Laboratory BITTA, Bologna, Italy; 4 IRCCS Rizzoli Orthopedic Institute, Laboratory of Preclinical and Surgical Studies, Bologna, Italy; 5 Biology and Genetics Unit, Department of Biopathology and Medical Biotechnology, University of Palermo, Palermo, Italy; 6 Institute of Biomedicine and Molecular Immunology (IBIM), National Research Council, Palermo, Italy

**Keywords:** 3D cell culture system, Osteosarcoma, Spheroids, Scaffolds

## Abstract

Osteosarcoma (OS) is the most common primary malignant tumor of bone, which preferentially develops lung metastasis. Although standard chemotherapy has significantly improved long-term survival over the past few decades, the outcome for patients with metastatic or recurrent OS remains dramatically poor. Novel therapies are therefore required to slow progression and eradicate the disease. Furthermore, to better understand the cellular and molecular mechanisms responsible for OS onset and progression, the development of novel predictive culture systems resembling the native three-dimensional (3D) tumor microenvironment are mandatory. ‘Tumor engineering’ approaches radically changed the previous *scenario*, through the development of advanced and alternative 3D cell culture in vitro models able to tightly mimic the in vivo tumor microenvironment.

In this review, we will summarize the state of the art in this novel area, illustrating the different methods and techniques employed to realize 3D OS cell culture models and we report the achieved results, which highlight the efficacy of these models in reproducing the tumor milieu. Although data need to be further validated, the scientific studies reviewed here are certainly promising and give new insights into the clinical practice.

## Background

Osteosarcoma (OS), is the most common primary malignancy of bone that develops preferentially in metaphyseal areas of rapidly growing long bones (distal femur, proximal tibia and humerus), where the balance between osteoblast, osteoclast and mesenchymal precursor activities is altered [1]. It is characterized by spindle cells of mesenchymal origin depositing immature osteoid matrix and shows a peak incidence from puberty until young adulthood (10–30 years) [2]. The abnormal precursors invade the primary ossification center and are subject to environmental stimuli able to modulate tumor progression within the growth plate (GP) [3]. To date, surgery associated to standard chemotherapeutic protocols represents the first-line treatment for most OS, with a significant increase in survival rates [3]. Despite numerous advances in clinical management, OS is still a highly aggressive tumor with poor prognosis when metastases occur (20% of patients), commonly targeting lung and other bones [4]. The outcome for these patients remains unfavorable and the research of novel pharmacological approaches for the treatments is mandatory [5].

Several studies attributed the origin of OS to chromosomal instability, including several genetic alterations, such as mutations in the tumor suppressor gene P53 and/or the retinoblastoma gene RB1, which are master cell cycle regulators linked to mesenchymal stem cells (MSC) differentiation [2, 6]. The loss of function in P53 and/or RB1 of MSC or MSC-derived osteogenic cell represents the major factor for OS development, with the concomitant influence of proper signals within the tumor microenvironment [7].

To investigate the mechanisms underlying OS pathogenesis, progression and resistance to treatments, two-dimensional (2D) cell cultures model were set-up and used [8]. It was found that cell-cell and cell-matrix interactions have a key role in tumor morphogenesis and cancer progression. However, the use of 2D approaches to study cancer cells and tumor process [9] failed to explain tumor cell biology, because they did not mimic real tumor macrostructure and did not reflect the complexity (tumor-stroma interactions) and heterogeneity (tumor variants) of the tumor microenvironment [10–12]. In addition, even if these 2D systems were able to study the ability of tumor cells to grow, they did not adequately take into account the physiological three-dimensional (3D) environment, failing to provide information about cell–cell (3D network between tumor cells with stromal, immune and endothelial cells) and cell-extracellular matrix (ECM) interactions, as well as cell surface receptors expression, growth factor synthesis and physical and chemical conditions [12, 13]. Conversely, several experimental studies highlighted the capabilities of in vitro 3D culture systems to recapitulate the characteristic of tumor tissues [14].

To date, the knowledge acquired in the field of tissue engineering pointed out the concept of “tumor engineering” as a “complex culture model that mimics in vivo tumor microenvironment in order to study the dynamics of tumor development and progression, and to develop clinically relevant models to target cancer and cancer stem cells” [15, 16]. This review on 3D in vitro models of OS tries to highlight the recent improvements of these techniques, which might represent a good compromise between the limitations of 2D in vitro models and the complexity of in vivo models that need to take into account various ethical and technical aspects such as animal housing, handling and welfare, the high costs of experimental procedures, and the compliance with scrupulously international legislation and regulations.

## Search strategies

The following literature search was carried out in the MEDLINE database (PubMed research engine) to identify studies employing 3D in vitro models of OS, including original articles in English from January 2007 to August 2017. The keywords were: (((((“Osteosarcoma”[Mesh] OR (“osteosarcoma”[MeSH Terms] OR “osteosarcoma”[All Fields]))))) AND ((((“Spheroids, Cellular”[Mesh] OR Spheroids? [All Fields]) OR 3D[All Fields]) OR 3d[All Fields] OR three-dimensional[All Fields]))) AND ((((((culture?) OR co-culture?) OR model?) OR system?))). Two reviewers manually assessed the title and abstract of collected references.

## Results and discussion

**Fig. 1:**
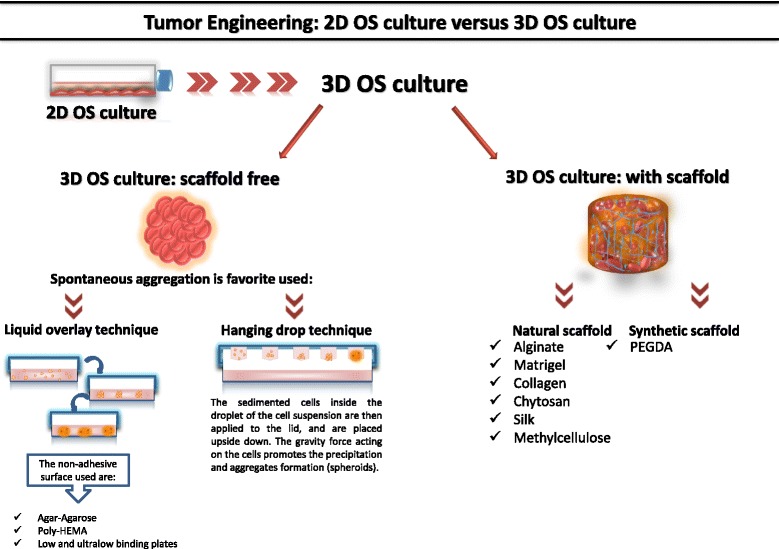
Schematic representation of techniques for 3D OS cultures

An initial literature search retrieved 107 articles. The resulting references were selected for supplementary analysis, based on the title and abstracts and 56 articles were considered eligible for the review. Complete articles were then reviewed to establish whether the publication met the inclusion criteria and 36 articles were recognized eligible for the review. By considering 3D models reported for OS studies in the selected bibliography, we stratified the papers according to: 3D cell culture system - scaffold free and 3D cell culture system - with scaffold (Fig 1).

### 3D CELL culture system - scaffold free

**Fig. 2:**
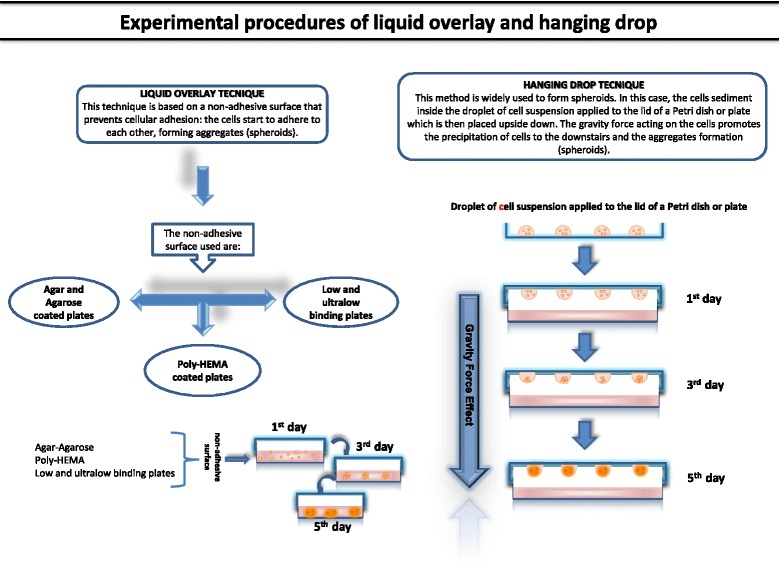
Comparative representation of the main techniques used to realize 3D OS spheroids cultures

3D OS culture scaffold-free can be generated through a spontaneous aggregation process [17], facilitated by, liquid overlay [18, 19] or hanging drop techniques [20, 21] (Table 1 and Figure 2).

**Table 1:** Techniques for 3D OS culture-scaffolds free

LIQUID OVERLAY TECNIQUE	METHOD	CELL LINES	REF.
Agar and Agarose-coated plates	To realize 3D culture: - 5.0x10^5^ cells/well seeded in tissue culture plates (Nunc) covered with 3% agar dissolved in RPMI plus heat-inactivated 10% FBS (37°C, 5% CO_2_).	MG-63; human fibroblast cell line CRL 7124	[22]
	To realize 3D culture: - plates were coated with 1.5% (wt/vol) agarose solution (0.15 g agarose and 10 mL PBS). The solution was heated to facilitate agarose dissolution. 70μl of hot agarose solution was pipetted into each well. The solution was kept on a hot plate during the well coating process to prevent premature gelatinization, and then allowed to cool for 20 minutes. - 100 μL of the cell suspension was added to each agarose-coated well. After 72 hours, cells resulted aggregate.	U2OS; MDA-MB-231	[10]
Poly-HEMA-coated plates	Gibbs et al. method was used to form 3D culture: - 60,000 cells/well are seeded in poly-HEMA-coated plates. - Serum-free DMEM/F12 medium with 1% of methylcellulose supplemented with 1% penicillin/streptomycin, 20 nM progesterone, 100μM putrescine, 1% insulin-transferrin-selenium A supplement, 10ng/ml bFGF, and 10ng/ml hrEGF. 3D spheroids were obtained after 7 days in culture.	Human MNNG/HOS	[23]
Low and Ultralow binding plates	To realize 3D culture: - cells were seeded in NanoCulture ® plates (NCPs; 96-well, low-binding; SCIVAX Corporation) and incubated at 37°C, 5% CO_2_.	HS-Os-1; NOS-1; SaOS-2; SJSA-1; 143B; HOS; HuO9; KHOS/NP; MG-63; MNNG-HOS; NOS-10.	[18]
	To realize 3D culture: - 2×10^4^ U2OS cells resuspended in 100μl RPMI medium with 10% FBS and penicillin-streptomycin (1% 100 units/mL), were plated on Corning® Spheroid Microplates. - The plates were centrifuged at 1000 RPM x 5 min, and incubated for 72 hrs.	U2OS; A549, C2C12, DU145, F9, GH3, HeLa, HEp2, NIH3T3, PA317, SH-SY5Y, and 293T	[19; 24]
	Zhang et al. method was used to form 3D cultures: - 10×10^3^ OS cells were grown in Ultra Low Attachment plates, - 2 mL serum-free MEM or McCoy’s medium with 20 ng/mL bFGF, 20 ng/mL EGF, B27, 100 μg/mL gentimycin and antibiotic-antimycotic. - Spheroids are obtained after 7 culture days.	143B; MNNG/HOS; SaOS-2; MG-63; U2OS; SJSA-1	[25]
	To realize 3D cultures: - 1.56x10^4^ cells/ml were seeded on ultralow attachment plates (Corning). - Serum-free William’s E medium with GlutaMAX supplemented with putrescine (100 mM), sodium selenite (30 nM), transferrin (25 mg/ml), insulin (20 mg/ml), hr-bFGF (10 ng/ml) and EGF (10 ng/ml) and incubated at 37°C, 5% CO_2_. - Additional growth factors (100 mg/ml) were added to the media every other day.	Canine osteosarcoma cells; KTOSA5 and CSKOS.	[27]
	3D OS spheroids were obtained maintaining OS cells in non-adherent round-bottom 96-well plates (Greiner bio-one) for 7, 14 and 19 days.	Canine OS cells (D17)	[20]
	To realize 3D cultures: - 5000 cells/well cells plated in ultralow attachment plates (Corning). - RPMI-1640 supplemented with B27 Supplement, 10 ng/mL human EGF, and 10 ng/mL human bFGF (fresh aliquots were added every other day), and incubated at 37°C and 5% CO_2_. - After culture for 2 weeks, spheroids reached > 50 μm in size.	MG63; U2OS; 43B	[29]
HANGING DROP TECHNIQUE	METHOD	CELL LINES	REF.
	To realize 3D cultures: - Cells were seeded into Gravity PLUS^TM^ plates (InSphero), at a density of: SaOS-2, 2000 cells/40μl (drop volume); HOS, 2500 cells/40μl; MG-63, 250cells/40μl; OS cells from patients, 2500 cells/40μl (day 0). - DMEM/Ham’s F-12 supplemented with 10% horse serum, 25 mM HEPES buffer and 1× penicillin/streptomycin was used.	SaOS-2; HOS; MG-63; human osteoblastic OS cells.	[21]
	To realize 3D cultures: - 60 cells/μL were seeded to get 400 μm diameter spheroids at the beginning of the treatment with the compound. - After 48 h, the compacted spheroids were transferred to an agar coated plate. - 200 μL of DMEM plus 10 % FBS were added to each well.	MG63	[32]
	To realize 3D cultures: - MG63 cells were seeded into Gravity PLUS^TM^ plates (InSphero), at a density of 1x10^4^ cells/40μl (drop volume) to obtain spheroids with a diameter of 400 μm. - DMEM supplemented with 10% FBS, 1mM Na-pyruvate, 2mM glutamine, 250 U ml^-1^ fungizone, 10 U ml^-1^ penicillin, 100μg ml^-1^ streptomycin was used. - 7 μL of culture medium were added to each well every 3-4 days.	MG63, HUVEC	[33]

## Liquid-overlay

The liquid-overlay method prevents cells to adhere to the vessel surface, which can be coated with non-adherent material, such as agar and agarose, or poly-hydroxyethyl methacrylate (HEMA), or using commercially low or ultralow binding plates [11].

### Agar and agarose

Indovina et al. studied the effects of hypoxia on adhesion and spreading of MG-63 spheroids formed by liquid-overlay technique, using plates covered with 3% agar in order to mimic the relationship between OS cells and microenvironment. They observed that hypoxia condition increased MG-63 spheroids adhesion to tissue culture plates, collagen type I and fibronectin, or to fibroblast in co-culture. All these systems were employed to mimic the relationship between OS cells and microenvironment. Moreover, hypoxic conditions induced over-expression of two key integrins in MG-63 spheroids - the alfa chains of fibronectin receptor (α5) and collagen receptor (α2), promoting tumor adhesion and spreading. In addition, they observed a concomitant reduction in fibronectin expression in 3D spheroid, favoring tumor cells spreading and migration [22]. Another interesting study was carried out by Charoen et al. who evaluated drug and drug-device combinations using a multicellular spheroids cancer model realized by spontaneous aggregation and growth of OS cells on agarose-coated plate. To better mimic ECM in vivo properties they also embedded these spheroids in a collagen matrix. The authors observed that the application of this model: (a) supported the production of spheroids of controllable size and scalable for high throughput applications; (b) reproduced cell-cell and cell-ECM interaction, as observed in vivo; (c) developed in vitro and in vivo solid tumor mass with a necrotic core and a metabolically active outer cellular layer. Furthermore, OS spheroids embedded in collagen exhibited a differential drug response in terms of efficacy depending on different delivery methods (bolus or within expansile nanoparticles), which did not arise in monolayer cell cultures. Notably, in vitro results using 3D model were quite similar to those achieved in vivo [10].

### Poly-HEMA

An interesting study was set-up by Martin et al. to identify and characterize cancer stem cell (CSCs) populations in a human OS cell line and to explore their role in the responsiveness to chemo- and radio-therapy by culturing them in poly-HEMA-coated plates. The CSC population isolated showed stem-like properties: (a) formation of 3D OS spheroids clone under anchorage-independent conditions, using poly-HEMA-coated plates; (b) expression of MSC surface markers CD73, 90, 103 and the three lineage differentiation capability; (c) expression of Oct4 and Nanog, key transcription factors necessary to maintain self-renewal and pluripotency; and (d) ability to retain self-renewal and generate differentiated progeny observing serial passages under selective culture conditions. The authors showed the chemo- and radio-resistance capacity of spherical clones compared with parental cells, which was attributed to the increase of ABC transporters (such as PgP and BCRP pump) that mediate drug efflux (such as doxorubicin, cisplatin and methotrexate). Additionally, it was demonstrated that OS CSC drug resistance increased through various mechanisms, i.e. the ability to enter in quiescent state, reducing the energy requirement and, consequentially, the cellular dividing rate, the enhanced capacity to repair potential lethal damage induced by ionizing radiation, reducing ROS production and, finally, being less susceptibile to apoptosis compared with parental cells, suggesting that sarcospheres possess highly activated basal DNA repair mechanisms [23].

### Low and ultralow binding plates

Arai et al. adopted low binding plates to realize 3D OS spheroids and compare the different behavior of monolayer and 3D OS spheroids principally in terms of chemoresistance after treatment with doxorubicin. They reported that 3D OS cells were more chemoresistant in comparison to conventional monolayer cultures [18]. The proteomic analysis of 11 OS cell lines highlighted 17 protein spots whose expression resulted differently regulated in OS spheroid cultures compared to monolayer conditions. Four of these proteins were associated with doxorubicin resistance induced by spheroid-formation and, among them, cathepsin D was identified. This had already been associated with an anti-apoptotic effect in other tumor models and used as biomarker of poor prognosis in breast cancer [18]. Similarly, Beak et al., adopting the same technique to realize a 3D model, confirmed that OS cells were more chemoresistant in 3D compared to 2D culture. The authors used a 3D culture system to monitor the effects of doxorubicin [19] and cisplatin [24] on several tumor cells, including U2OS OS cell line organized in spheroids. Analyzing the morphological (growth and shape) and energetic (ATP production) change of spheroids in real-time, they concluded that the core densification of OS spheroids, during treatment with increasing drug doses, led to a reduction in drug permeability without altered metabolic activity. These observations suggested the role of ECM within the aggregates as barrier to limit drug permeation. The densification of the spheroid core, promoted also the increase of drug IC50 in 3D compared to 2D models. This aspect highlighted the higher cytotoxicity of 2D culture in comparison to 3D system and underlined the necessity to use 3D models for drug screening [19, 24]. Rainrusso et al. isolated tumor-initiating cells (TIC) from six OS cell lines by functionally labeling slowly dividing/quiescent cells using the fluorescent dye, and obtained TIC spheroids using ultralow binding plates. The functional capacity of TIC to generate bone tumors was demonstrated not only in vitro, through the identification of surface specific markers, but also analyzing their behavior through in vivo models obtained by orthotopic injection in immunodeficient mice. The genomic profile of OS TIC cells revealed deregulation of pathways involved in bone/skeletal development, with the over-expression of insulin growth factor-1 (IGF-1) and indian hedgehog (IHH) factors, this latter resulting particularly enriched. Furthermore, OS TIC revealed increased migratory properties, thus underling the role of this cell population in the metastatic process. Finally, the authors argued that a deep comprehension of TIC behavior might contribute to improve treatments, combining cytotoxic drugs and new agents, selectively targeting these slowly dividing/quiescent cells. This property was correlated with OS TIC resistance to most chemotherapeutic agents, which target rapidly dividing cells [25]. Another interesting study was carried out by Pang et al. who isolated a subpopulation of cells with stem-like properties in a primary canine OS, resistant to conventional chemotherapy. They used ultralow binding plates to realize anchorage-independent culture in order to form spheroids. The study revealed that CSCs played a critical role in OS response to therapy; microarray analysis (spheres versus adherent cells) identified an increased expression of Aldehyde dehydrogenase 3A1 (ALDH3A1) and Cyclooxygenase-2 (COX-2) in the CSCs population. ALDH3A1 in canine CSC cells had a detoxifying role, thus contributing to the resistance to chemotherapeutic drugs. On the other side, as also reported in another study, COX-2 inhibition reduced tumor growth and progression in human OS [26]. However, in this study COX-2 inhibition had no effect on CSC growth, or resistance to chemotherapy in 3D culture, but a significant effect on the sphere-forming capacity in daughter adherent cells was observed instead. Authors concluded that CSCs played a critical role in assessing the response to therapy of OS patients, while COX-2 may play a role in tumor formation and maintenance [27]. OS canine cells were employed also by Gebhard et al. who used the spontaneous aggregation and growth of these cells on ultralow binding plate, reproducing native in vivo OS architecture through autologous matrix production (collagen type, vimentin and osteocalcin). Their study highlighted that the size of spheroids was a conditioning parameter to reproduce the characteristic of solid tumor mass. Indeed, only the smallest spheroids reproduced a necrotic core in their inner mass, which was surrounded by a rim of viable and proliferative cells. In addition, they proposed this model as highly reproducible, and assumed to be suitable for drug and hypoxic testing assays [20]. Using the same technique, Guo and colleagues realized 3D OS spheroids to identify a regulator of OS CSC activity. They observed that the level of miR-335 was downregulated in OS CSC compared to their differentiated counterparts; notably, this specific miRNA was reported to be correlated with stem properties of OS cells [28]. Indeed, reducing the miR-335 level in three OS cell lines, they observed the increase of: a) their capacity to form spheroids; b) expression of CSC markers (CD117 and Stro-1); c) invasion and migratory properties; d) expression of POU5F1 and Sox2, involved in maintaining OS CSC fate. These changes led to a consequent increase of chemoresistance in comparison to traditional chemotherapy. They concluded that miR-335 replacement treatment reduced the activity of OS CSC, suggesting that it could be a potential agent able to eradicate CSCs [29].

## Hanging drop

To date, the most diffuse method to realize spherical micro-masses from immortalized and primary cell lines is represented by the hanging drop technique [30]. Micromasses are also generated by multi-cell type co-cultures that do not require any synthetic materials support, but that take advantage of gravity [31]. Rimann et al. used the hanging drop method to overcome the limits of 2D culture which does not reflect the dynamic 3D microenvironment of the native tumor and offer a limited assay time window to verify drug efficacy [21]. The study developed 3D OS micromasses using different human OS cell lines (HOS, MG63, SaOS2) and primary cells in order to better recapitulate the heterogeneity of OS. They observed that drug resistance was associated to several micromass morphologies, reflecting different drug uptake kinetics, closely related to the tightness of 3D structures. In addition, they affirmed that drug resistance was associated with the tumor microenvironment, acting as a physical barrier leading to cell adhesion-mediated drug resistance [21]. Leon et al., adopted the same technique to study the behavior of OS cells using 3D multicellular tumor spheroids (MCTS) that simulated the cytoarchitecture of a solid tumor. They observed that cell growth was linear until the 2nd day of culture and cell proliferation correlated with the increase of micromass volume. However, after 5 days of culture no variations in spheroids size occurred, and the edges of MCTS seemed more regular and uniform. This similarity with solid tumors led the authors to promote MCTS as a good preclinical model to screen the effects of the complex oxidovanadium (IV) -flavonoid chrysin (VOchrys). They showed that VOchrys (100 μM) decreased the viability of OS MCTS and displayed antitumor actions, since it altered the shape of spheroids. The authors confirmed the in vitro data, observing a similar effect on an in vivo xenograft mice model [32]. Chaddad et al. proposed an in vitro 3D vascularized tumor model, leaving 3D OS spheroids obtained after 5 days in the hanging drop to interact with 2D human endothelial cells, to closely mimic the tumor microenvironment. The expression levels of the angiogenic factors VEGF, ICAM1 and CXCR4 were analyzed in several cell culture conditions such as: MG63 OS cells and HUVEC cells grown in monolayer, MG63 spheroids and HUVEC 2D/MG63 3D co-culture. Notably, MG63 cells in 3D culture expressed more bone proteins and VEGF than in monolayer, while tumor cell-derived VEGF attracted endothelial cells in 2D/3D co-culture conditions, creating a vascular network. Furthermore, MG63 spheroids cultured on HUVEC monolayer expressed more VEGF, ICAM1 and CXCR4, when compared with MG63 monolayer*.* The authors observed a co-localized signal of vessel-like structures markers (CD31 and collagen IV) in specific areas of the spheroid mass in 2D/3D co-culture, and explained it with the increased expression of angiogenesis-related factors associated to the creation of tubular-like structures [33].

### 3D CELL culture system - with scaffold

The scaffolds commonly used to realize 3D culture include naturally derived matrices and synthetic materials. The characteristics responsible for cells migration, proliferation and aggregation include chemical composition, shape, structure, and porosity [34]. The natural scaffolds are represented mainly by typical components of ECM such as collagen, elastin, laminin, fibrin, gelatin, matrigel or hydrogels derived from natural sources, such as chitosan, silk, alginate, hyaluronic acid, heparin and chondroitin sulfate [35]. Natural scaffolds are able to favor cell interaction properties, adhesion and signaling, and they are also biodegradable [35]. As for synthetic scaffolds, they are mainly represented by polyethylene glycol (PEG), polyvinyl alcohol (PVA), polylactide-co-glycolide (PLG), and polycaprolactone (PCL), hydrogel or solid matrix, and ceramics such as calcium phosphate biomaterials, bioactive hydroxyapatite and tricalcium phosphate or their associations (HA/TCP) [36, 37]. The primary advantage of these natural and synthetic materials is their high biocompatibility and responsiveness to cell adhesion without any modification [38]. Among these natural and synthetic matrices, those used for 3D OS culture are alginate, gelatinous protein mixture secreted by Engelbreth-Holm-Swarm (EHS) mouse sarcoma cells (Matrigel™ or Cultrex® BME) with or without association with collagen I, chytosan, silk, methylcellulose, bacterical cellulose and polyethylene glycol diacrylate (PEGDA) (Table 2).

**Table 2:** Techniques for 3D OS cultures with scaffolds

NATURAL SCAFFOLD	METHOD	CELL LINES	REF.
Alginate	To realize 3D cultures: - 4 x10^6^ cells/ml were encapsulated in 1.2% low-viscosity sterile alginate in 0.15 M sodium chloride (NaCl). - The cell suspension in alginate solution was slowly pushed through a 21-gauge needle and dropped into a 102 mM CaCl_2_ solution. Beads were allowed to polymerize in this solution for 10 min before two consecutive washes with 50 ml of 0.15 M NaCl. - Maintained in DMEM supplemented with 10% FBS and gentamicin (50 μg/ml), and incubated at 37°C and 5% CO_2_. Media were changed daily.	Dunn; LM8	[5, 39]
Matrigel and Collagen	HOS and MG-63 cells were mixed with matrigel and cultured for 48 h.	HOS; MG-63	[40]
	To realize 3D cultures: - 300μl of 3D BME (Cultrex) scaffold were seeded into the plates and transferred in incubator at 37°C for 30 min to promote gel formation. - 2.0x10^4^ cells were seeded on top of the thick gel in each well. - Maintained in DMEM (supplemented with 10% FBS) and incubated at 37°C and 5% CO_2,_ for up to 14 days. Media were replaced every 3 days.	3AB-OS CSCs	[41]
	To realize 3D OS cultures: - 10^5^ OS cells were mixed with 3% Matrigel in complete medium. - Cellular suspensions were loaded into culture plates, allowed to polymerize at 37°C, and then overlaid with complete media.	RF379L; RF1044; RF43	[42]
	To realize 3D OS cultures: - 2.4 mg/ml collagen gel solution was prepared for the bottom layer, while a 1.2 or 2.4 mg/ml solution was used for the upper layer. - After gel polymerization in the bottom layer, cells were suspended in collagen gel solution and added to the dish, then transferred to 37°C for 60 min to polymerize, and covered with culture media.	Dunn; LM8	[46]
	To realize 3D cultures: - 6.0x10^4^ cells were mixed with 50μl neutralized collagen I solution and left for 2h while polymerization took place. - 3D fibrillar collagen I gels were prepared by adding 50 μL neutralized collagen I solution (7:1:1:1 of each 3 mg/mL) collagen I in 0.2% acetic acid, 10x serum-free medium, 1.0mM Hepes, pH 7.3, and 0.33mM NaOH, to each well.	OHS	[47]
	To realize 3D cultures: - 250 μl of Matrigel or Type I collagen was dropped onto glass coverslips and allowed to polymerize for 1 h at 37°C. - 7.5×10^5^cell/ml was transfected and seeded on top of the gels, maintained in DMEM 10% FBS and incubated at 37°C and 5% CO_2_.	MG63	[48]
	To realize 3D cultures: - gel isolated from mouse tails was dissolved in 0.013 mol/L HCl (final concentration 5 mg/mL). Type I collagen solution (200 μl) was mixed with 674 μl H_2_O (on ice). The mixture was added to 26 μl NaOH (0.1 mol/L). The new mixture was added to 100 μl of 10x RPMI 1640 medium, and 5μl of the mixture was added onto each slide and incubated at 37°C for 1h until the gel solidified. - 5x10^5^ LM8 cells/mL seeded on Type I collagen gel, supplemented with a basal medium with or without ZA.	LM8	[49]
	To realize 3D cultures: - 100,000 cells/ml were added to the unpolymerized collagen solution for proliferation assay and 30,000 cells/ml for migration assay. - Collagen Type 1 stock solution was diluted to 3 or 4 mg/ml, mixing equal volumes of neutralizing buffer (100 mM Hepes in 26 PBS, pH 7.3) with PBS. - To polymerize, gels were placed for 2h in the incubator (37°C, 5% C02), resulting in cell-embedded collagen gels. Medium was replaced daily.	U20S; MCF7	[50]
	To realize 3D cultures: - OS cells were directly added to the cooled hydrogel solution, composed by Matrigel (Beckton Dickinson) and collagen I. - 14.5μL of gel-cell suspension (2000 cells/well) were transferred in culture plate using a robotic liquid handler (CyBio Selma 96/60). - After polymerization (30 min at 37°C, 5% CO_2)_, growth medium was added.	MOS; U2OS	[51, 52]
Chytosan	To realize 3D cultures: - composite matrices were made by freezing and lyophilizing a suspension of chitosan (medium-molecular-mass chitosan, 250 kDa; Aldrich) and nanohydroxyapatite (nHA powder; Aldrich) as described by Thein-Han & Misra (2009). The dimensions of pores were variable, with a mean cross-section of ~100 mm. - 10^6^ cells were plated per matrix and their growth was followed.	U2OS; SaOS-2	[53]
Silk	To realize 3D cultures: - 3% (w/v) Silk fibroin, derived from Bombyx mori silkworms silk solution, was placed in 55mm diameter petri dishes, frozen at 20°C and freeze dried overnight to form porous silk sponges. - The sponges were then soaked in 90% methanol to convert the silk into the b-sheet structure and washed with distilled water. - 0.5x10^6^ cells were suspended in DMEM 10% FBS, 1% penicillin/streptomycin and infused into the silk or nanohydroxyapatite-coated silk scaffolds. - The scaffolds were incubated at 37°C, 5% CO_2_.	143.98.2	[54]
	To realize 3D cultures: - 0.5x10^6^ U2OS cells or U2OS cocultured with immortalized fibroblasts were seeded in the silk scaffold. - Cultured for 7 days.	U2OS; HPV-16 E6/E7; HUVEC	[55]
	To realize 3D cultures: - 0.5x10^6^ of OS cells were suspended in 30μl complete culture medium (DMEM 10% FBS, 1% penicillin/streptomycin) and injected into silk scaffolds.	SaOS2; U2OS,143.98.2	[56]
Methylcellulose	To realize 3D cultures: - 200 cells/well were mixed with 20% methylcellulose and were seeded into round-bottom Ultra-Low Attachment plates, in 100μl DMEM-F12 containing 20 ng/ml epidermal growth factor (EGF), 20 ng/ml basic fibroblast growth factor (bFGF), 10 ng/ml hepatocyte growth factor (HGF), 10 % B27, 2 % bovine pituitary extract (BPE). - Spheroids were incubated at 37 °C and 5 % CO_2,_ and harvested at various time points for RNA isolation or drug testing as described below.	RD; HT1080; SW872; HOSS1	[57]
Bacterical cellulose	To realize 3D cultures: - Bacterial cellulose sheets were cut with a biopsy punch to 0.32 cm^2^ and sterilized in a steam autoclave (at 121°C for 20 min). - 100ul of CSC cells (10^5^cells/mL) suspension was seeded onto the drained scaffolds. - Scaffolds were incubated (37°C, 5% CO_2_) for 1h to allow cell attachment, and then 1mL of fresh McCoy’s 5A medium, 2.5% FBS and 2 mM L-glutamine were added onto each scaffold. - The scaffolds were placed in their respective hypoxic or control experimental conditions.	SaOS-2	[14]
SYNTHETIC SCAFFOLD	METHOD	CELL LINES	REF.
PEGDA	To realize 3D cultures: - The hydroxyl end-groups of the PEG macromer was made to react with acryloyl chloride to produce PEG diacrylate (PEGDA). - 100 mg PEGDA macromer was dissolved in a photoinitiator solution (10 mg Irgacure-2959 in 1 mL PBS) sterilized by filtration. - Next, tumor cells were uniformly suspended in gel precursor solution by gentle mixing to a final density of 0.3-2.0x10^6^ cells/mL. - The cell-suspended gel precursor solution was crosslinked by UV irradiation, with peak wavelength of 365 nm. - After crosslinking, gels were cut into disks and incubated in stem cell medium to form tumor spheres.	MDA231; MCF7; HCT116; AGS; U2OS.	[58]

## Natural scaffold

### Alginate

Akeda et al. proposed a 3D OS model, realized by using alginate beads, to study the capability of OS cells to maintain the malignant phenotype. To set-up this 3D system, they compared the growth of murine OS cell lines: the parental Dunn and its derivative line LM8 that exhibited a higher metastatic potential to the lung. They observed that both cell lines grew in vitro and in vivo in alginate beads and that LM8 cells had an higher capacity to detach from the alginate environment in comparison to parental Dunn cells, thus reflecting the original malignant potential of this cell line [5]. Subsequently, Nishimura et al., adopting the same culture method, investigated whether the formation of OS pulmonary metastasis was decreased by inhibiting the action of nuclear factor-kappa B (NF-kB). They directly transfected LM8 cells encapsulated in alginate with NF-kB decoy oligodeoxynucleotide (ODN) and showed that the DNA-binding activity of NF-kB by LM8 cells was inhibited. Tumor growth was not altered by transfection, but mRNA levels of vascular endothelial growth factor (VEGF) and intercellular adhesion molecule - 1 precursor (ICAM-1) - were reduced and this aspect correlated with the malignancy and the metastatic potential of OS. The inhibitory role of NF-kB decoy ODN on the number of pulmonary metastases was further confirmed by an in vivo alginate beads transplantation model [39].

### Matrigel and collagen

Suzuky et al., using a 3D matrigel model, analyzed the role of pericellular ECM in the intracellular uptake of NF-kB decoy oligonucleotides to reduce invasion and motility of OS cell lines induced by tumor necrosis factor alfa (TNFα). They observed the inhibitory effects of ECM on cellular uptake of labeled oligonucleotides. However, after enzymatic removal of pericellular matrix, the uptake markedly recovered in MG-63 cells. These results supported the role of pericellular matrix in disturbing the uptake of NF-kB decoy and underlined how the modification of matrix composition might increase the efficacy of exogenous oligonucleotides treatment for OS [40]. Adopting a 3D matrigel culture model, Di Fiore et al. studied the behavior of 3AB-OS CSC in vitro after miR-29b-1 replacement. Analyzing these cells in comparison to parental MG-63 OS cells, they observed that miR-29b-1 was highly downregulated. The replacement of miR-29b-1 in 3AB-OS CSC impaired cell proliferation, acting on its putative targets (CCND2, E2F1 and E2F2) involved in cell cycle regulation and DNA synthesis, thus producing smaller spherical masses compared to untreated cells. They found that miR-29b-1 overexpression decreased spheroid-forming ability, colony formation and the level of stemness markers such as Oct3/4, Sox2 and Nanog, while it did not influence the migratory and invasive capabilities of 3AB-OS cells. Furthermore, they demonstrated that miR-29b-1 induced doxorubicin, cisplatin and etoposide chemosensitivity versus doxorubicin, cisplatin and etoposide, decreasing the expression level of anti-apoptotic genes BCl-2 and IAP-2, with subsequent reduction of the cellular apoptosis. Finally, they suggested that miR-29b-1 could be a novel potential therapeutic agent in OS treatment [41]. Techavichit et al. adopted a 3D OS matrigel culture using highly and low metastatic OS cell lines in order to confirm the role of secreted frizzled-related protein 2 (sFRP2) in tumor aggressiveness [42]. The role of sFRP2 protein was associated with Wnt signaling pathway [43] and several studies reported the key role of this protein in tumor progression and aggressiveness [44, 45]. This study, using gain and loss of function experiments, highlighted how the altered sFRP2 expression had no effect on tumor cell proliferation, but significantly affected the migratory and invasive potential of OS cells, both in vitro and in vivo [42]. Differently from the above-mentioned studies, Tanaka et al. adopted a 3D collagen model to study the colony formation step in lung, and the contribution of the microenvironment stiffness on tumor cells behavior. The authors focused their attention on the metastatic cells capability to detach themselves from tumor mass and escape through the endothelial barrier to reach the lung. To analyze the effect of microenvironment stiffness on tumor cells, this study employed a 3D culture using collagen I gel at different concentration in order to mimic the lung environment. They observed that LM8 metastatic cells in 3D culture, realized with several stiffness conditions mimicking the lung environment (150 Pa), had the capacity to form bigger colonies and to grow more rapidly than Dunn parental cells. Unfortunately, the mechanism by which LM8 preferred lung-mimicking conditions compared to Dunn is still unknown [46]. To investigate the effect of cell-collagen I interactions on the synthesis and activation of matrix metalloproteinase-2 (MMP-2), Elenjord et al. used in vitro models characterized by 3D fibrillar and 2D monomeric OS cell culture. MMP-2 is considered the major MMP enzyme involved in ECM remodeling, whose dysregulated activity is involved in cancer. The interaction of OS cells with two form of collagen I, promoted opposite effects on cellular synthesis and activation of MMP-2, as well as the synthesis of membrane type 1 MMP (MT1-MMP), and the decrease of tissue inhibitors of MMP -1 (TIMP-1). These factors are regulated by the small calcium-binding protein (S100A4), also associated with tumor progression. In this study the interaction of OS cells with 2D monomeric collagen or 3D fibrillar collagen, determined ECM remodeling, acting on S100A4-induced pathway that down- or up-regulate TIMP-1 expression. Results underlined that the structure of the surrounding matrix components has relevant effects on OS cells, promoting invasion and metastatic process [47].

Zhang et al. investigated the role of the VE-cadherin in OS cells grown on 3D matrigel and 3D Collagen type I system. OS cells showed the vasculogenic mimicry capacity to obtain nutrients and oxygen, mimicking some functions of endothelial like-cells to favor tumor progression. They explained this behavior through inhibition of VE-cadherin gene expression by using a specific siRNA in OS cells, observing a consequent reduction of angiogenic sprout and endothelial-like networks. The authors established that VE-cadherin was involved in the trans-differentiation process of OS cells into endothelial-like cells and function. These models provided an important tool for the study of angiogenic phenotype and vasculogenic mimicry, comparing matrigel matrix versus collagen I. Results showed that collagen I was better in mimicking physiological conditions, because it is a neutral element without growth factors addition. Finally, the authors defined their method as optional for the observation of early OS invasion, angiogenic process and efficiency of anti-vascular formation therapy [48].

To inhibit OS cells vasculogenic mimicry process, Fu et al.*,* grew the highly metastatic LM8 OS cell line on 3D collagen type I matrix and tested for the first time the effect of Zoledronic Acid (ZA, bisphosphonate family). They observed that OS cells, grown on a thin layer of type I collagen gel, attached the extracellular matrix and showed behaviors similar to in vivo growth of tumor cells. In comparison to tumor cells embedded in 3D collagen type I matrix, OS cells cultured on collagen type I layer expressed their vasculogenic mimicry capabilities 12 h after cell seeding. Treating the 3D culture with ZA induced the inhibition of vasculogenic mimicry effect of LM8 OS cells, probably due to the block of RhoA translocation from the cytosol to the membrane. This pharmacologic effect determined the disruption of F-actin polymer, resulting in the alteration of cytoskeletal structure, with consequent dramatic reduction of microvilli and filopodia, thus having an effect on vasculogenic mimicry development [49].

Fallica et al., analyzed the behavior of OS cells embedded in 3D collagen gel reconstituted with two different concentrations. They observed the alteration of cellular behavior and response to Phosphatide Inositolo-3 Kinase (PI3K) pathway inhibition. PI3K/AKT pathway is an important intracellular signaling cascade, which affects cell growth, migration, protein expression and survival. In many cancer types, this pathway is deregulated and investigated as potential target for new anti-cancer drugs. The authors employed the dual kinase inhibitor cell-permeable PI103 that exert effects on PI3K and its mammalian target of rapamycin (mTOR). Results showed that it was sufficient to culture U2OS cell line in 3D collagen matrix reconstituted with two different concentrations to alter the activation of PI3K/Akt pathway, specifically observing the reduction of cellular proliferation and migration. After treatment with PI103, drug response of U2OS cells was affected by ECM properties (i.e. mechanical matrix stiffness). Indeed, higher concentrations of collagen I resulted in a more resistant population of U2OS cells [50]. Differently, Baranski et al., using a 3D culture system realized by mixing cells with hydrogel containing matrigel and collagen I, carried out a kinase inhibitor screen to detect candidate targets for human OS. They identified the mitogen-activated protein kinase (MEK) inhibitors as potential therapeutic targets in cells with constitutive extracellular signal-regulated kinases (ERK) activation. Moreover, MEK inhibition promoted apoptosis on several OS cell lines in comparison to other resistant cell lines [51]. Finally, they also detected that low concentrations of Chk1 inhibitors led to an effective sensitization of 3D OS culture to low concentrations of doxorubicin, thus reducing chemoresistance effects [52].

### Chytosan

Dey et al. confirmed the utility of 3D model in OS studies growing OS cells in monolayer and in 3D chitosan-nanohydroxyapatite composite matrices and demonstrated that the growth of OS cells in 3D bone like matrix alters their susceptibility to adeno-associated virus (AAV)-2. They showed that the susceptibility was correlated with the lack of an innate antiviral response by OS cells. When OS cells were infected with AAV-2 in 2D or 3D cultures, the authors found that OS cells, grown in 3D culture, were less susceptible to viral infection. In this 3D system, OS cells expressed alkaline phosphatase, a specific marker of bone cell differentiation, associated with a reduction of Rep protein and capsid. In conclusion this study revealed that differentiated OS cells were less permissive for AAV-2 infection, probably due to the variability of expression of cell membrane receptors [53].

### Silk

Tan et al. used a porous silk sponge scaffold to realize an OS 3D model [54] and showed that the secretion profiles of OS cells grown on 3D silk scaffolds were different in comparison to 2D culture. 3D culture conditions regulated the expression of basic fibroblast growth factor (bFGF), hypoxia-inducible factors (HIF)-1α, VEGF-A and interleukin (IL)-8, closely reflecting what occurs in mouse xenograft tumors models. This is probably due to the contribution of hypoxia developed within the 3D architecture of porous silk scaffolds similarly to what happens in an in vivo model [54]. Subsequently, the authors also evaluated the effect of 3D architecture and tumor-stroma interaction on the secretory profile of angiogenic factors in U2OS cells, in both 3D monoculture and 3D co-culture with immortalized fibroblast. The 3D co-culture of U2OS with fibroblasts led to a significant up-regulation of IL-8 and VEGF-A mRNA level, which favored HUVECs migration in transwell system co-culture, in comparison with 3D monoculture of U2OS. Conversely, they showed that the treatment with monoclonal antibody against VEGF-A (Avastin) and IL-8 produced different results on HUVEC migration, demonstrating that anti-IL8 therapy had more efficacy on 3D U2OS/fibroblast co-culture compared to 3D U2OS monoculture, inhibiting HUVEC migration. These experiments confirmed the relevance of 3D tumor co-culture models as a preclinical model for drug testing, also promoting combined therapy against IL8 and VEGF to be used as antiangiogenic drugs [55]. In their latest scientific work, Tan et al., investigated the effects of 3D OS silk architecture on cellular proliferation and drug sensitivity and observed how 3D culture influences the proliferation rate of OS cells, bringing a reduction in comparison to 2D culture model. The transition from 2D to 3D culture determined the arrest of cell cycle in G1, the down regulation of several factors (cyclin B1, E2F1 and RhoA) involved in cell cycle progression and, finally, the upregulation of p21, a negative regulator of cell cycle. The arrest of 3D OS culture in G1 made OS cells more chemoresistant to cell cycle specific chemotherapeutic agents, such as doxorubicin, while no effect was reported for cisplatin, a cell cycle non-specific agent. These data were confirmed by: a) cytotoxicity assays, which allowed to assume that the exponential growth of OS cells in 2D culture made it more sensitive to doxorubicin; b) gene expression analysis, which did not show any increase of multi drug resistance 1 (MDR1) expression levels. Notably, data obtained underlined how the 2D culture method provided altered results on the evaluation of chemotherapeutic drug dosages in comparison to 3D culture. Together, the results obtained by Tan et al. highlighted that the 3D silk model mimicked better the real behavior of the tumor mass, did not affect the expression of angiogenic factors, appeared to be inert like conventional culture substrates and provided information about real chemotherapy effects [56].

### Methylcellulose

Bai et al. employed the methylcellulose matrix to recreate the 3D environment of sarcoma including OS, and to analyze the mechanisms of chemo- and radio- resistance. They evidenced that methylcellulose made a better disintegration of 3D mass (by trypsinization method), useful for carefully evaluating cancer cells proliferation. Compared to 2D, cells in 3D cultures maintained stable exponential growth without reducing proliferation. Furthermore, gene expression analysis showed that several genes involved in tumor adhesion (cadherin), gap junction formation (connexin), ECM production (collagen, fibronectin, integrin, proteoglycan, laminin), chemotherapy resistance (ABC transport and antiapoptotic proteins) were up-regulated in a 3D model. Consequentially, analyzing the response to several chemotherapeutic agents and X-ray irradiation, tumor cells in 3D culture appeared less sensitive to drug-induced apoptosis. For these reasons, the authors explained the chemo- and radio- resistance effects to genetic rearrangement induced by 3D culture conditions. The study underlined the requirement to mimic physiological microenvironments in vitro, indicating in the 3D cell culture model a predictive tool for screening drugs for OS therapy [57].

### Bacterial cellulose

The effects of hypoxia on the maintenance of CSC in OS were investigated by Gorgun et al. by using an OS 3D culture model consisting of an innovative biosynthetic scaffold formed by bacterial cellulose. They showed that bacterial cellulose with its intrinsic characteristic (high purity, strength, and hydrophilicity) was useful not only to mimic tumor ECM and its microenvironment, but also to study CSC behavior under hypoxic conditions. Hypoxic 3D culture had no negative effects on CSC viability, but it supported the expression of several CSC markers, i.e. OCT3/4, Nanog, SOX2 as well as CD133, in comparison to normoxic conditions. These results confirmed the capability of bacterial cellulose to recreate hypoxic conditions, accurately mimicking in vivo conditions of OS niche and preserving CSC pluripotency [14].

## Synthetic scaffold

### PEGDA

Jabbari et al. studied the effects of 3D matrix stiffness on the maintenance of CSC properties in several tumor cell lines, including OS cell line. Cells were encapsulated in polyethylene glycol diacrylate (PEGDA) hydro-gel, a 3D matrix modifiable in stiffness. They compared cellular growth under equal conditions (cell density, gel structure and mesh size, gel composition, culture medium composition), revealing that the optimum modulus for growth and expression of CSC markers by tumor-spheres grown in the PEGDA gel, depended on the origin of cancer cells tissue. In particular, they demonstrated that the optimum matrix stiffness for growth and marker expression of CSC sub-population of OS cancer cells was 50 kPa (stiffness); in fact, in this condition, the number of OS tumor-spheres was considerably increased. Furthermore, they evidenced that during the first days of incubation in 3D culture with optimum gel stiffness modulus, several cell lines, including OS cells, showed an increased expression of epithelial mesenchymal transition (EMT) markers (as YAP/TAZ transcription factors). This aspect indicated that a sub-population of cancer cells had undergone EMT by acquiring the CSC phenotype. The expression level of EMT markers underlined that CSC sub-population resided within a niche with optimum stiffness, correlating with cancer cells tissue of origin, and this model reproduced it [58].

## Conclusion

The awareness that the tumor is not merely a mass of transformed and proliferating cancer cells, but that it possesses intrinsic heterogeneity and complexity, able to support and mediate tumor resistance mechanism, together to the need to find new drugs able to overcome chemoresistance phenomena, led to the development of various advanced experimental techniques that better mimic in vivo conditions, strongly different from the commonly used experimental in vitro conditions [15]. The knowledge acquired in the field of tissue engineering and applied to the study of cancer cells biology, gave birth to the concept of “tumor engineering” as a complex culture model that strictly mimics “in vivo*”* tumor microenvironment, i.e. tumor development and progression, allowing to develop clinically relevant models, able to target cancer and cancer stem cells [16].

The current review underlines the relevance of the introduction of “tumor engineering” techniques to study the behavior of OS cells in a 3D cell culture model. The analysis of the methods reported in literature showed that all 3D OS culture methods (scaffold free or with scaffold) represent useful tool to recapitulate the complex tumor environment and, among them, spheroid seems to be the most used and suitable system, able to favor micro-mass formation in vitro [59]. The spheroid model is considered to be the most physiologically relevant 3D model for tumor studies [60]. This model is characterized by hypoxic regions and necrotic centers, representing a valid model able to mimic tumor microregions or micro-metastases [17]. In addition, it is also valuable as "*in-vivo* like 3D cell culture model" to study chemotherapeutic response or gene therapy approaches [5, 30]. Indeed, as widely highlighted in this review, when OS cells grow in 3D “spheroids”, spontaneously or in scaffolds, cell-cell and cell–ECM interactions contribute to modify cell morphology and behavior, closely resembling physiological characteristics [36].

The spheroids realized with “3D cell culture system: scaffold free” provide high performance, [60] and allow a more accurate characterization and analysis of OS, and OS-CSC behavior. In addition, these spheroids might lead to the identification of novel therapeutic targets and could also have a key role in the evaluation of sensitivity to chemotherapeutic agents in order to eradicate the cancer stem cell niche [23, 25, 29, 61]. Notably, despite the cytoarchitecture of 3D model determines chemoresistance, due to the lower capability of drug to penetrate [62], the application of the “3D cell culture system without scaffold” allowed to obtain data comparable to the in vivo model, adopting cheaper techniques and following two of the 3R principles – replacing and reducing – related to the use of animals for scientific purposes [63]. In addition, the application of “3D cell culture system with scaffold” gives further possibility to control chemical composition, shape, structure, porosity and stiffness of the 3D matrix, influencing cell-cell interaction and proliferative and migratory capabilities of tumor cells [34, 64]. The prediction of human response to drugs represents an emerging challenge in clinical practice. Data obtained by 2D cell culture are notoriously less reliable because the cell monolayer does not reflect the real three-dimensional tumor microenvironment, which is also difficult to realize with animal models due to the multiple pathophysiologic variables to take into account [60].

To our knowledge, no 3D in vitro model fully reproduce the complexity and heterogeneity of OS by taking into account the tumor microenvironment, but the 3D approach could realistically simulate the microenvironmental physiology of tumors if more aspects were to be considered: 1) the intimate interaction with non-neoplastic cells inside the tumor microenvironment, including fibroblasts, endothelial and immune system cells 2) the intrinsic heterogeneity of the tumor [65].

The present review described and highlighted the conditioning of 3D OS cells by ECM interaction and the efficacy of spheroids models to strictly reproduce the native tumor milieu. Only Chaddad and Tan described 3D interaction between OS cells with endothelial cells and fibroblast in co-culture conditions, analyzing the behavior of OS cells in relationship to the vascular niche. The skeletal vascular microenvironment was identified as “bone-marrow vascular niche”, which is involved in controlling the fate of tumor cells, active and dormant, and can influence multiple steps within the metastatic cascade [66].

Unlike OS, 3D heterotypic spheroid models composed of tumor cells, fibroblasts, and immune cells, were much more investigated in other tumors. The 3D heterotypic culture system was developed to investigate reciprocal interactions of T_REG_ lymphocytes and NK cells with luminal and basal phenotype breast cancers [67]. Furthermore, the 3D heterotypic spheroid model seems to provide novel efficacy tools for in vitro evaluation of cancer immunotherapy agents, investigating immune cells infiltration and drug targeting [68]. Other studies tested 3D heterotypic culture systems investigating the reciprocal interaction among cancer and endothelial cells, the latter being able to create well established tubular networks and luminal structures inside the tumoral spheroid [69]. Patient-derived cells in 3D cultures represent a great advantage for addressing intra-tumoral heterogeneity and predict drug efficacy for cancer treatment; for instance, 3D cell culture samples from patients with metastatic colorectal cancer were used to compare the genetic diversity of patient-derived tumor organoids with the original tumor biopsy; notably, genetic analysis highlighted that organoids reflect the metastasis they derived from, supporting organoid cultures as ex vivo platforms to personalize anticancer treatment [70].

However, much effort must be taken to assure reproducibility, high throughput analysis, compatible readout techniques, and automation to establish standardized and validated homotypic or heterotypic 3D cell culture models in clinical trials with a large cohort of patients, thus attributing a clinical-predictive value for personalized medicine. These aspects might greatly improve OS management, making 3D OS model an attractive tool for researchers and clinicians.

## Abbreviations

2D: Two-dimensional
3D: Three-dimensional
ALDH3A1: Aldehyde dehydrogenase 3A1
COX-2: Cyclooxygenase-2
CSCs: Cancer stem cells
ECM: Extracellular matrix
GP: Growth plate
HA/TCP: Hydroxyapatite and tricalcium phosphate
ICAM-1: Intercellular adhesion molecule-1
IGF-1: Insulin growth factor-1
IHH: Indian hedgehog
MCTS: Multicellular tumor spheroids
MMP-2: Matrix metalloproteinase-2
MT1-MMP: Membrane type 1 MMP
ODN: Oligodeoxynucleotide
OS: Osteosarcoma
PCL: Polycaprolactone
PEG: Polyethylene glycol
PEGDA: Polyethylene glycol diacrylate
PI3K: Phosphatide Inositolo-3 Kinase
PLG: Polylactide-co-glycolide
PVA: Polyvinyl alcohol
S100A4: Small calcium-binding protein
sFRP2: Secreted frizzled-related protein 2
TICs: Tumor-initiating cells
TIMP-1: Tissue inhibitors of MMP -1
TNFα: Tumor necrosis factor alfa
VEGF: Vascular endothelial growth factor
VOchrys: Oxidovanadium (IV) complex with the flavonoid chrysin
ZA: Zoledronic Acid
